# Gastrointestinal symptoms and CPAP-related aerophagia A questionnaire study.

**DOI:** 10.1007/s11325-025-03360-w

**Published:** 2025-05-23

**Authors:** Anne Hillamaa, Timo Mustonen, Adel Bachour

**Affiliations:** 1 Heart and Lung Center, Helsinki University Hospital, Helsinki, Finland; 2 Clinical Physiology Unit, Helsinki University Hospital, Helsinki, Finland; 3 Sleep Unit, Heart and Lung Center, Helsinki University Hospital, Helsinki, Finland

**Keywords:** Obstructive sleep apnea, Continuous positive airway pressure, Aerophagia, C-aerophagia, Gastrointestinal symptoms

## Abstract

**Purpose:**

Aerophagia is a functional gastrointestinal disorder including swallowing air, repeated belching, and disturbing symptoms of air retention in the gastrointestinal tract. Aerophagia can also occur during continuous positive airway pressure (CPAP) therapy. Pressurized air can leak into the stomach causing belching, abdominal distention, discomfort, and flatulence, among other symptoms. There are few studies on CPAP-related aerophagia (C-aerophagia), and the reported prevalence ranges from 8.28 to 16% Our aim was to evaluate aerophagia-related symptoms before and with CPAP therapy.

**Methods:**

A total of 2004 patients began CPAP therapy at the Helsinki Sleep Apnea policlinic during 2015–2019. A randomly selected sample of 1059 patients were sent a questionnaire to assess symptoms possibly related to aerophagia. Symptom severity was evaluated with a Visual Analogue Scale (VAS).

**Results:**

We received 324 responses. The most disturbing symptom was flatulence, with VAS increasing from a median of 24 before CPAP to 34 during CPAP (*p* ≤ 0.001). Dry mouth increased from 16 to 31 (*p* ≤ 0.001), heartburn decreased from 12 to 10 (*p* ≤ 0.001), and belching decreased from 12 to 9 (*p* = 0.018). A total of 29 patients abandoned CPAP therapy. Symptoms of aerophagia were the main cause of abandoning CPAP for 3 patients.

**Conclusions:**

Most patients who reported symptoms of aerophagia with CPAP therapy were already symptomatic before CPAP initiation. Only flatulence and dry mouth increased slightly with CPAP. Although changes in symptoms were very mild overall, in rare cases (1%) symptoms can be very disturbing and even lead to abandoning CPAP therapy. For clinical practice, it is important to assess whether symptoms of aerophagia are actually related to and worsened by CPAP therapy to avoid unnecessary interventions.

**Supplementary Information:**

The online version contains supplementary material available at 10.1007/s11325-025-03360-w.

## Introduction

Some individuals experience a troublesome pattern of swallowing air and repeated belching, along with disturbing symptoms of air retention in the gastrointestinal tract. This is called aerophagia, a specific functional gastrointestinal syndrome. The Rome III consensus stated that the diagnostic criteria for aerophagia in adults must include troublesome belching at least several times a week and that swallowing of air is objectively observed [1]. Aerophagia has since been removed from the Rome IV criteria for adults and it is only found in the functional gastrointestinal disorders of children and adolescents [2]. Aerophagia is a very common functional gastrointestinal disorder, but patients rarely seek medical attention [3].

Obstructive sleep apnea (OSA) is a disorder associated with increased daytime sleepiness, cardiovascular disease, and reduced quality of life [4–6]. Continuous positive airway pressure (CPAP) therapy is the first-line therapy and the most effective treatment for moderate and severe sleep apnea. CPAP delivers pressurized air to the upper airways, splinting them open. Clinical improvement during CPAP therapy is directly related to a major reduction in respiratory events during sleep [7].

CPAP therapy, despite being effective, has limitations. Patient compliance is not without problems, and approximately one third of patients may fail therapy [8]. Among the reasons for poor compliance are side effects associated with CPAP, such as nasal dryness, interface (mask) air leak, pressure intolerance, and claustrophobia [9, 10].

Aerophagia may also occur during CPAP therapy (C-aerophagia). CPAP therapy increases intrathoracic and esophageal pressure that may increase air leak into the stomach, especially when swallowing. The ingested air can collect in the gastrointestinal system, causing belching, abdominal distention, discomfort, and flatulence, among other symptoms [11]. There are few studies on C-aerophagia, and its mechanisms are poorly understood. The prevalence of aerophagia during CPAP has been reported to range from 8.28 to 16% [12, 13] and up to 52% of OSA patients on CPAP have been reported to suffer symptoms of aerophagia [11].

Very recently, Fukutome has published an interesting paper about C-aerophagia using newly developed diagnostic criteria [14]. The prevalence of C-aerophagia was estimated to be 7.2%.

Suspicion of aerophagia relies on clinical symptoms. Aerophagia without CPAP has a prevalence of 23%.^3^ For diagnosis, we recently suggested esophageal pH-impedance measurement simultaneously with polysomnography as the gold standard method to confirm CPAP-related aerophagia [15].

We suspect that the prevalence of C-aerophagia may be underestimated. Our clinical impression suggests that aerophagia is a side effect that is difficult to tolerate and may be a significant reason for why patients abandon CPAP. Finding practical solutions to reduce aerophagia and relieve the patient discomfort may help improve CPAP adherence.

The main purpose of this questionnaire study was to evaluate the aerophagia-related symptoms of the gastrointestinal tract before and after beginning CPAP therapy.

## Methods

### Subjects

We used a hospital procedural code registry to identify a total of 2004 patients that were referred to our sleep clinic for CPAP initiation between the years 2015 and 2019. Among them we randomly selected a sample of 1059 patients due to time and budget restrictions. Randomization was performed with SPSS software (IBM^®^ SPSS^®^ Statistics Version 29.0.0.0).

All patients were on automated positive airway pressure (APAP) therapy, except for some patients who had significant central apneas who were on CPAP. The studied population reflected inhabitants of Helsinki, of whom more than 90% are of Caucasian origin.

### Questionnaire

We created a questionnaire (Supplement 1) to assess symptoms of the gastrointestinal tract that may be caused by or related to C-aerophagia. These included abdominal bloating, gas being trapped in the stomach, heartburn, belching or burping, abdominal pain, nausea or vomiting, flatulence, diarrhea, a disturbing feeling of fullness or loss of appetite, drooling or excessive salivation, or dry mouth. The respondents were asked about their symptoms prior to beginning CPAP therapy and with CPAP therapy. Symptom severity was evaluated with a Visual Analogue Scale (VAS), measured in millimeters, zero meaning no symptoms and a score of 100 meaning the worst possible symptoms. For those who had continued with CPAP, we assessed CPAP daily use. Moreover, we asked about the measures patients had taken to relieve possible gastrointestinal symptoms related to CPAP use.

### Statistical analysis

Data analysis was performed using SPSS. For all tests, significance level was *p* < 0.05. The Chi square test and independent t-tests were used to assess differences between responders and non-responders. The nonparametric Wilcoxon signed-rank test was used to assess intragroup differences prior to and with CPAP use. Spearman’s correlation test was used to assess correlations. We used a Bonferroni test correction to reduce the instance of a false positive in multiple comparison.

### Ethics

This study was approved by the HUS Regional Committee on Medical Research Ethics. All participants provided written informed consent.

## Results

A total of 1059 patients were selected randomly for the study. As 30 subjects had no valid address, the questionnaire was sent to 1029 patients. We received 332 responses, yielding a response rate of 32%. There was a statistically significant difference in sexes between responders and non-responders. Of the responders, 31,2% were female and 68,8% were male. Of the non-responders, 39,4% were female and 60,6% were male. Therefore, males were more likely to answer the questionnaire (*p* = 0,013).

On average, the responders were 61,6 years old, while the non-responders were 55,9 years old. There was a statistically significant difference in the age of responders and non-responders (*p* < 0,001).

**Fig. 1:**
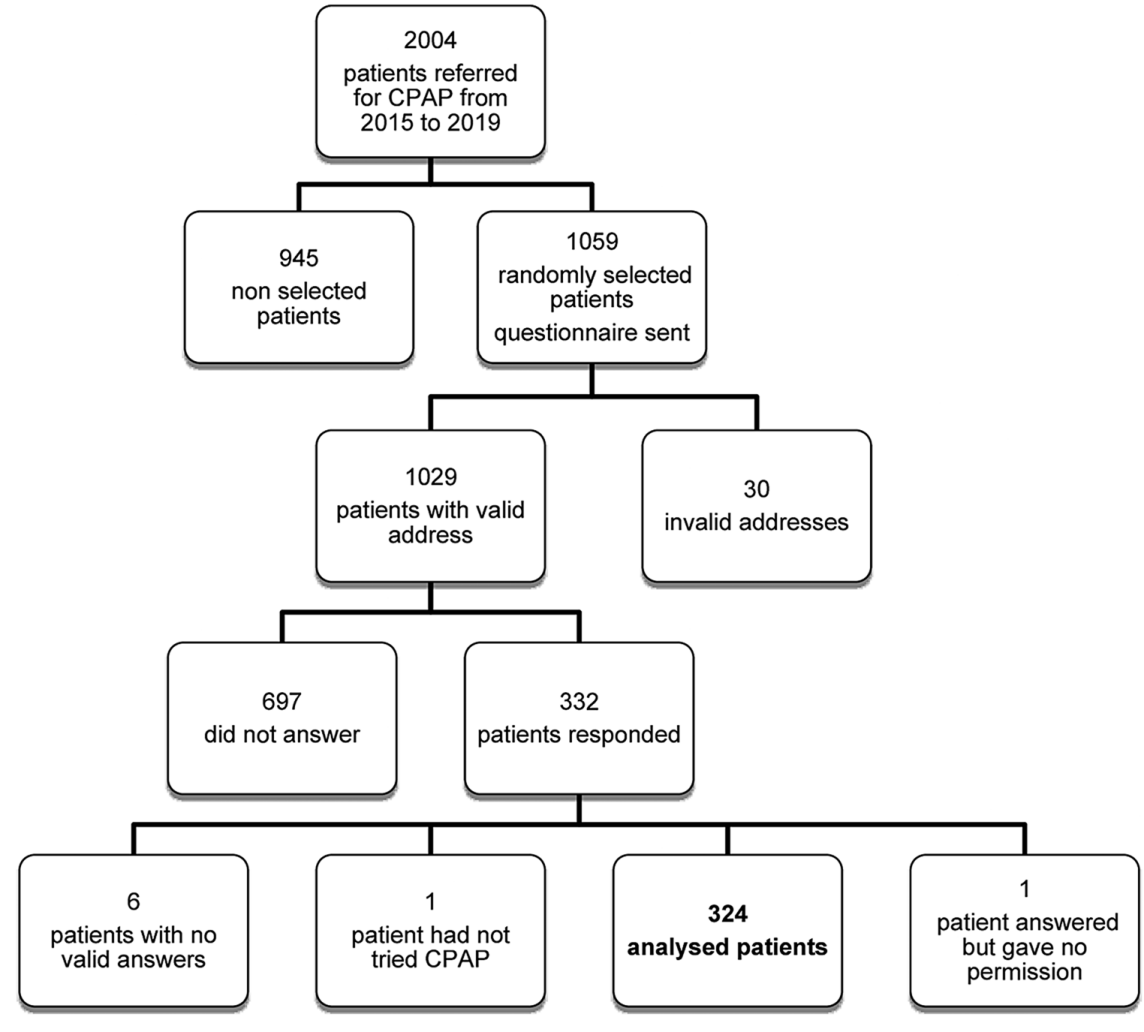
Flow chart of the study.

Of the returned questionnaires, 324 were analyzed; 6 patients returned the questionnaire but left it empty with no answers or personal information and one patient did not give permission to use their response in the study. One patient reported not having tried CPAP but instead having been treated with oral appliance therapy (Fig. 1).

The mean (range) age was 62 (20–89) years. We asked patients about their CPAP use. Eleven responders were unsure or their answer was missing. Of the valid answers, 284 patients (89.6%) were still using CPAP. Twenty-nine patients (9.1%) had stopped using CPAP. There were multiple reasons for abandoning CPAP therapy; the most common was difficulty adjusting to the treatment (9 responders) and having sleep problems with CPAP (5 responders). Three (10%) responders who abandoned CPAP therapy had done so because of gastrointestinal symptoms.

**Fig. 2:**
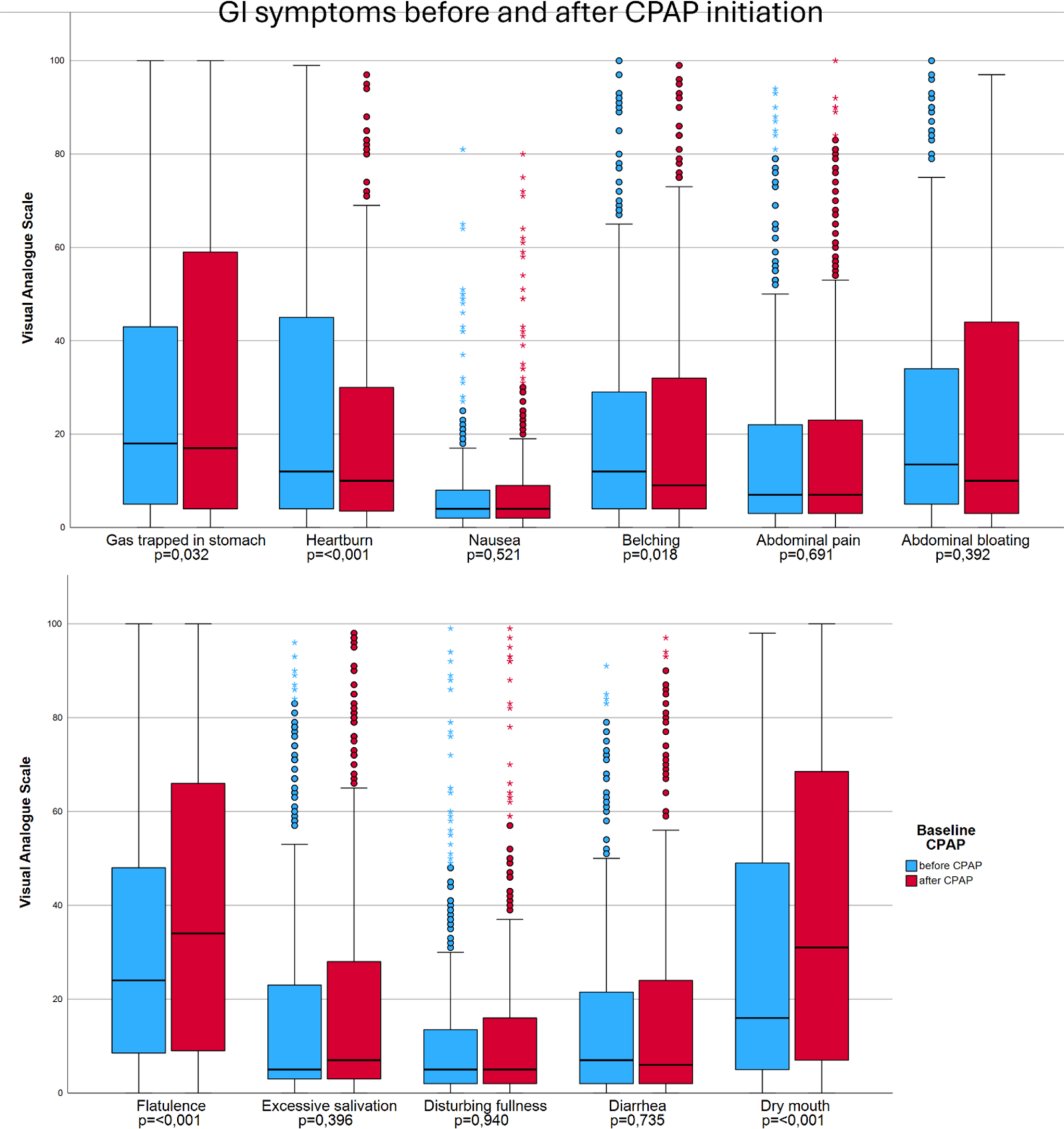
Boxplot showing change in gastrointestinal symptoms with CPAP therapy. There was a significant increase in dry mouth and flatulence with CPAP therapy.

Gastrointestinal symptoms were very mild to moderate before CPAP therapy (Fig. 2). The most disturbing symptom was flatulence, with VAS increasing from a median of 24 before CPAP to 34 during CPAP (*p* ≤ 0.001). Dry mouth increased from 16 to 31 (*p* ≤ 0.001) with CPAP. Some symptoms were relieved with CPAP therapy; heartburn decreased from 12 to 10 (*p* ≤ 0.001) and belching decreased from 12 to 9 (*p* = 0.018).

Women presented with more gastrointestinal symptoms before CPAP use. With CPAP, there was no significant change between men and women, with the exeption of dry mouth. On average, women experienced a more significant increase in dry mouth than men (18 vs. 7, *p* ≤ 0.001).

A total of 22 patients did not report their CPAP usage hours. On average, CPAP was used for 6,6 h per night.

A Spearman’s correlation was used to explore the relationship between hours of CPAP used and gastrointestinal symptoms. A weak, positive and statistically significant correlation was found between CPAP usage and belching, r(322) = 0.17, *p* = 0,002. Other symptoms showed no statistically significant correlation with CPAP usage.

No correlation between age and symptom severity was found.

Of the patients who recognized novel or increased GI symptoms with CPAP therapy, 54% presented with GI symptoms within one month of therapy, 31% of patients had symptoms emerging between one month and one year, and 15% after one year of therapy.

A total of 63% of responders tried some measures to relieve their CPAP-related GI symptoms. The most common was changing sleeping position, which was attempted by 45.1%. Other measures included stopping eating and drinking at least 1 h before bed (19.4%), raising the head of the bed (17%), and decreasing the CPAP maximal pressure (9.9%). A total of 20.1% tried other means, which most commonly included drinking water, adjusting the mask, or raising the humidity setting on the CPAP machine.

## Discussion

We studied several gastrointestinal symptoms and their relation to CPAP therapy by a questionnaire. Our main finding was that GI symptoms existed before CPAP initiation and were changed slightly by CPAP therapy.

Only two GI symptoms increased significantly with CPAP. The first was flatulence, which may be explained by C-aerophagia (swallowing pressurized air). In this regard, our results agree with Fukutome. The second symptom was dry mouth, which is usually alleviated with CPAP therapy except for patients that present with air leak through the mouth [16]. Although these increased significantly with CPAP, clinically this increase was minor. Regarding the diagnostic symptoms of C-aerophagia used by Fukutome, we did not see changes in abdominal bloating, abdominal pain and belching.

CPAP therapy increases the pressure gradient between the stomach and the lower esophagus. Therefore, heartburn and belching decreased with CPAP therapy, as expected.

In Fukutome’s study, the diagnostic criteria were restricted to only a few symptoms. We looked widely at several GI symptoms that might be associated with aerophagia. As it is unknown how long the symptoms of C-aerophagia can last, we also accepted symptoms experienced by patients throughout the day and not just during CPAP use.

The second major finding was that even though the CPAP-related GI symptoms were mild overall, about 10% of the patients who abandoned CPAP therapy did so because of GI symptoms. This equals 1% of our study population. This highlights the limited influence of GI symptoms on CPAP adherence.

The third major finding was that most C-aerophagia symptoms appeared during the first month of CPAP use. CPAP follow-up should be active when beginning treatment [17]. We fully agree with this conclusion and recommend active observation and support during the early stage of CPAP therapy.

Regarding the relationship between CPAP usage and gastrointestinal symptoms, we ran a correlational analysis. The only statistically significant correlation was found for belching and the correlation was weak. We propose exploring this relationship in another study.

Our study has some strengths. All patients used the same CPAP brand equipped with humidifiers, had an optimal CPAP interface, and were followed up regularly to minimize CPAP therapy side effects. Our sleep apnea unit covers 95% of all sleep apnea patients in our area, a population of 650 000 individuals. Therefore, there was no bias in patient selection. CPAP equipment was offered without extra charges, therefore no financial factors interfered with CPAP therapy. The majority of our patients used CPAP for over 4 h per day, therefore our results reflect well the results of CPAP therapy.

Our study also has some limitations. The questionnaire response rate was relatively low. This could be explained by the somewhat long questionnaire. Patients with no GI symptoms may also have low motivation to respond. There were small but statistically significant differences between responders and non-responders. We received relatively more responses from men than women. Nevertheless, we could still detect an increase in dry mouth in women. The responders were slightly older than non-responders. However, we did not find any correlation between age and symptom severity. Moreover, we did not study the effect of CPAP mask or sleep apnea severity on GI symptoms. We also did not ask about the beneficial effects of some measures used to alleviate GI symptoms. This may be interesting to investigate in future studies.

When GI symptoms emerge or increase with CPAP therapy, C-aerophagy should be suspected and an esophageal pH and impedance measurement during PSG should be requested to confirm the diagnosis and avoid unnecessary interventions or treatments [15].

## Conclusions

Our data suggest that the previously reported prevalence of C-aerophagia may be overestimated, as we observed that gastrointestinal symptoms were common even before CPAP therapy. Only a few GI symptoms increased with CPAP therapy, such as flatulence and dry mouth, and this increase was moderate. Flatulence is likely due to C-aerophagia, but dry mouth with CPAP is usually related to air leak through the mouth. Only 1% of patients abandoned CPAP therapy due to C-aerophagia.

## Electronic supplementary material

Below is the link to the electronic supplementary material.

Supplementary Material 1

Supplementary Material 2

## Data Availability

The data analysed in this study are not openly available to others. The data can be accessed by applying for a research permit at HUS.
