# Supplementary material for: Gastrointestinal symptoms and CPAP-related aerophagia A questionnaire study
Source: Sleep Breath. 2025 May 23;29(3):197. doi: 10.1007/s11325-025-03360-w (PMC12101993; doi:10.1007/s11325-025-03360-w)
Supplement: Supplementary file 1 — Supplementary Material 1 [file 11325_2025_3360_MOESM1_ESM.docx]

HUCH, Heart and Lung Center

HUCH, Sleep Apnea Policlinic

**QUESTIONNAIRE ABOUT ABDOMINAL SYMPTOMS DURING CPAP TREATME
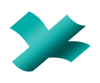
NT**

We are researching abdominal symptoms related to CPAP treatment at the Sleep Apnea Policlinic. CPAP-treatment is used to treat sleep apnea, a sleep-related breathing disorder. We kindly ask that you answer the following questionnaire.

**1. Have you tried CPAP treatment**  no [ ] yes [ ] I don’t know [ ]

**2. If yes, what year did you begin using CPAP treatment?**

__________________________________________________________________________________

**3. Do you still use CPAP?**  no [ ] yes [ ] I don’t know [ ]

**4. If you have stopped CPAP treatment, what was the reason?**

__________________________________________________________________________________

**5. How many hours on average do you use CPAP per day?**

**__________________________________________________________________________________**

**6. Have you had any of the following symptoms before you started CPAP treatment?**

Please mark an ”X” on the position that best describes your symptoms, for example:

x

not at all very much

**a. Abdominal bloating**

not at all very much

**b. Gas getting trapped in the stomach**

not at all very much

**c. Heartburn**

not at all very much

**d. Belching/burping**

not at all very much

**e. Abdominal pain**

not at all very much

**f. Nausea or vomiting**

not at all very much

**g. Flatulence**

not at all very much

**h. Diarrhea**

not at all very much

**i. Disturbing feeling of fullness or loss of appetite, especially in the morning**

not at all very much

**j. Excessive salivation**

not at all very much

**k. Dry mouth**

not at all very much

**7.** **Have you had any of the following symptoms after you started CPAP treatment?**

**a. Abdominal bloating**

not at all very much

**aa. How quickly does the bloating begin after starting CPAP use?**

______minutes ______hours

**ab. How long does the abdominal bloating last?** ______minutes ______hours

**b. Gas getting trapped in the stomach**

not at all very much

**c. Heartburn**

not at all very much

**d. Belching/burping**

not at all very much

**e. Abdominal pain**

not at all very much

**f. Nausea or vomiting**

not at all very much

**g. Flatulence**

not at all very much

**h. Diarrhea**

not at all very much

**i. Disturbing feeling of fullness or loss of appetite, especially in the morning**

not at all very much

**j. Excessive salivation**

not at all very much

**k. Dry mouth**

not at all very much

**8. How much do the previously mentioned symptoms disturb the use of CPAP therapy?**

not at all very much

**9. How quickly did the previously mentioned symptoms begin after starting CPAP therapy?**

______days ______weeks ______months ______years

**10a. Have you done anything to relieve symptoms related to CPAP treatment?**

[ ] changing position in bed

[ ] elevating the head of the bed

[ ] lowering CPAP pressure

[ ] stopping eating and drinking at least 1 hour before bed

[ ] something else, what?

_______________________________________________________________________________

**b. How much did these means help with your symptoms?**

not at all very much

**11. Do you allow the Sleep Apnea Policlinic to use you answers in medical research? The questionnaires will be processed anonymously. Participation is voluntary and will not affect your treatment. You have the right to cancel your participation at any time.**

yes, I give consent no, I do not consent to the use of my answers

[ ] [ ]

Name: ______________________________ Social security number: _____________________

**Thank you for your answer!**

Return to the following address:

Head physician Adel Bachour

Sleep Apnea Policlinic, Skin and Allergy Hospital, PL 160, 00029 HUS
