# Supplementary material for: Gastrointestinal symptoms and CPAP-related aerophagia A questionnaire study
Source: Sleep Breath. 2025 May 23;29(3):197. doi: 10.1007/s11325-025-03360-w (PMC12101993; doi:10.1007/s11325-025-03360-w)
Supplement: Supplementary file 2 — Supplementary Material 2 [file 11325_2025_3360_MOESM2_ESM.docx]

**KYSELY VATSAOIREISTA CPAP-HOIDON AIKANA
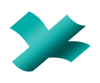
**

HYKS, Sydän- ja keuhkokeskus

HYKS, Uniapneapoliklinikka

Uniapneapoliklinikalla tutkitaan CPAP-laitehoitoon liittyviä vatsaoireita. CPAP-hoitoa käytetään unen aikana hoitamaan uniapneaan liittyviä hengityskatkoksia. Pyydämme ystävällisesti vastaamaan seuraavaan kyselyyn.

**1. Oletko kokeillut CPAP-laitehoitoa?**  ei [ ] kyllä [ ] en tiedä [ ]

**2. Jos vastasit kyllä, minä vuonna CPAP-hoito aloitettiin?**

__________________________________________________________________________________

**3. Käytätkö hoitoa edelleen?**  ei [ ] kyllä [ ] en tiedä [ ]

**4. Jos olet lopettanut CPAP-hoidon, mikä oli syynä?**

__________________________________________________________________________________

**5. Montako tuntia käytät CPAP-laitetta keskimäärin vuorokauden aikana?**

**__________________________________________________________________________________**

**6. Onko sinulla esiintynyt seuraavia oireita ennen kuin aloitit CPAP-hoidon?**

Laita rasti janalle siihen kohtaan, joka parhaiten kuvaa oireitasi, esimerkki:

x

ei lainkaan hyvin paljon

**a. Vatsan turvotus**

ei lainkaan hyvin paljon

**b. Ilman kertyminen vatsaan**

ei lainkaan hyvin paljon

**c. Närästys**

ei lainkaan hyvin paljon

**d. Röyhtäily**

ei lainkaan hyvin paljon

**e. Vatsakipu**

ei lainkaan hyvin paljon

**f. Pahoinvointi tai oksentaminen**

ei lainkaan hyvin paljon

**g. Ilmavaivat**

ei lainkaan hyvin paljon

**h. Ripuli**

ei lainkaan hyvin paljon

**i. Häiritsevä kylläisyyden tunne tai vähäinen ruokahalu, erityisesti aamuisin**

ei lainkaan hyvin paljon

**j. Kuolaaminen tai häiritsevä syljeneritys**

ei lainkaan hyvin paljon

**k. Suun kuivuminen**

ei lainkaan hyvin paljon

**7.** **Onko sinulla esiintynyt seuraavia oireita CPAP-hoidon aloittamisen jälkeen?**

**a. Vatsan turvotus**

ei lainkaan hyvin paljon

**aa. Kuinka nopeasti vatsan turvotus alkaa CPAP-laitteen käytön aloittamisesta?**

______minuuttia ______tuntia

**ab. Kuinka pitkään vatsan turvotus kestää?** ______minuuttia ______tuntia

**b. Ilman kertyminen vatsaan**

ei lainkaan hyvin paljon

**c. Närästys**

ei lainkaan hyvin paljon

**d. Röyhtäily**

ei lainkaan hyvin paljon

**e. Vatsakipu**

ei lainkaan hyvin paljon

**f. Pahoinvointi tai oksentaminen**

ei lainkaan hyvin paljon

**g. Ilmavaivat**

ei lainkaan hyvin paljon

**h. Ripuli**

ei lainkaan hyvin paljon

**i. Häiritsevä kylläisyyden tunne tai vähäinen ruokahalu, erityisesti aamuisin**

ei lainkaan hyvin paljon

**j. Kuolaaminen tai häiritsevä syljeneritys**

ei lainkaan hyvin paljon

**k. Suun kuivuminen**

ei lainkaan hyvin paljon

**8. Kuinka paljon edellä mainitut oireet häiritsevät CPAP-laitteen käyttöä?**

ei lainkaan hyvin paljon

**9. Kuinka nopeasti yllämainitut oireet alkoivat CPAP-hoidon aloittamisen jälkeen?**

______päivää ______viikkoa ______kuukautta ______vuotta

**10a. Oletko tehnyt jotain seuraavista helpottaaksesi CPAP-hoitoon liittyviä oireita?**

[ ] asennon vaihtaminen

[ ] sängyn päädyn kohottaminen

[ ] CPAP:n paineen alentaminen

[ ] syömisen ja juomisen lopettaminen vähintään tunti ennen nukkumaan menoa

[ ] muuta, mitä?

_______________________________________________________________________________

**b. Kuinka paljon edellä mainitut keinot auttavat oireisiin?**

ei lainkaan hyvin paljon

**11. Annatko Uniapneaklinikan henkilökunnalle luvan käyttää kyselyn vastauksia lääketieteellisessä julkaisussa? Kyselyt käsitellään nimettömänä. Osallistuminen on vapaaehtoinen, eikä vaikuta hoitoosi. Sinulla on koska tahansa oikeus peruuttaa suostumuksesi tutkimukseen.**

kyllä, annan luvan [ ] ei, kiellän vastausten käytön [ ]

Nimi: ______________________________ Henkilötunnus: _____________________

**Kiitos vastauksestasi!**

Palautus osoitteeseen:

Osastonylilääkäri Adel Bachour

Uniapneapoliklinikka, Iho- ja allergiasairaala, PL 160, 00029 HUS
